# Draft genome and transcriptome of *Nepenthes mirabilis*, a carnivorous plant in China

**DOI:** 10.1186/s12863-023-01126-5

**Published:** 2023-04-14

**Authors:** Yuan Gao, Hao-Bin Liao, Ting-Hong Liu, Jia-Ming Wu, Zheng-Feng Wang, Hong-Lin Cao

**Affiliations:** 1 Zhongshan Management Centre of the Natural Protected Area, Zhongshan, China; 2 Guangdong Provincial Key Laboratory of Applied Botany, Key Laboratory of Vegetation Restoration and Management of Degraded Ecosystems, South China Botanical Garden, Chinese Academy of Sciences, Guangzhou, China

**Keywords:** De novo assembly, Genome feature, Genome survey, Genome annotation, RNA-seq

## Abstract

**Objectives:**

*Nepenthes* belongs to the monotypic family Nepenthaceae, one of the largest carnivorous plant families. *Nepenthes* species show impressive adaptive radiation and suffer from being overexploited in nature. *Nepenthes mirabilis* is the most widely distributed species and the only *Nepenthes* species that is naturally distributed within China. Herein, we reported the genome and transcriptome assemblies of *N. mirabilis*. The assemblies will be useful resources for comparative genomics, to understand the adaptation and conservation of carnivorous species.

**Data description:**

This work produced ~ 139.5 Gb *N. mirabilis* whole genome sequencing reads using leaf tissues, and ~ 21.7 Gb and ~ 27.9 Gb of raw RNA-seq reads for its leaves and flowers, respectively. Transcriptome assembly obtained 339,802 transcripts, in which 79,758 open reading frames (ORFs) were identified. Function analysis indicated that these ORFs were mainly associated with proteolysis and DNA integration. The assembled genome was 691,409,685 bp with 159,555 contigs/scaffolds and an N50 of 10,307 bp. The BUSCO assessment of the assembled genome and transcriptome indicated 91.1% and 93.7% completeness, respectively. A total of 42,961 genes were predicted in the genome identified, coding for 45,461 proteins. The predicted genes were annotated using multiple databases, facilitating future functional analyses of them. This is the first genome report on the Nepenthaceae family.

## Objective

Carnivorous plants are special plants that can capture and digest insects and other animals through their pitfalls and traps. Nepenthaceae is one of the largest carnivorous plant families, and *Nepenthes* is the only genus in the family and contains 160–180 species [1]. *Nepenthes* species attract people with their special and beautiful pitcher traps, making them popular ornamental plants, which results in their overexploitation in nature. All *Nepenthes* species are currently listed under the Convention on International Trade in Endangered Species of Wild Flora and Fauna (CITES) [2]. *Nepenthes* species are also widely used in traditional medicine, making them multi-purpose species [3]. *Nepenthes* species grow in moist and nutrient-poor environments, showing varied ecological adaptations related to nutrient acquisition (such as different prey as substrates) [1]. They are also divergent into “Highland” and “Lowland” groups according to the different altitudes at which they grow [1]. Therefore, ecological divergence plays an important role in *Nepenthes* speciation. Moreover, hybridization is a common phenomenon in *Nepenthes* species, which causes uncertainty about their evolutionary relationships [1, 2]. Thus, evolutionary and taxonomic studies are highly required for *Nepenthes* species, especially when using their genome resources.

*Nepenthes mirabilis* is the most widely distributed species in the genus and can be found from southern China to Australia, Africa and some Pacific islands [4, 5]. In particular, it is the only *Nepenthes* species naturally distributed in China [4, 5]. Currently, genome resources for carnivorous plants are still lacking [6, 7], and no genomes have been reported for *Nepenthes* species. Here, we report the whole genome and transcriptome assemblies of *N. mirabilis* by using whole genome sequencing (WGS) and RNA-seq reads. The assemblies will be useful resources for comparative genomics and conservation of *N*. *mirabilis* and other carnivorous species. The data will help further understand the multifaceted adaptation of carnivorous plants at the genomic levels.

## Data description

One *N. mirabilis* sample was collected from Wuguishan (22°25’30.78” N, 113°26’16.37” E), Zhongshan City, China. Genomic DNA was isolated from its leaf tissues for WGS. Total RNAs were extracted from both its leaf and flower tissues. Standard genomic and trancriptomic 150 bp paired-end libraries were constructed and sequenced using the MGI DNBSEQ-T7 sequencing platform at GrandOmics Biosciences (Beijing, China). RNA-seq reads from leaves and flowers were combined to obtain the total transcriptome reads of *N. mirabilis*.

Multiple programs were used for genomic and transcriptomic sequencing data analysis. While performing these analyses, default parameter settings were applied, unless otherwise mentioned. The transcriptome reads were analyzed by the TransPi pipeline 1.3.0-rc [8], which included read filtering, *de novo* transcriptome assembly, and annotation. The raw WGS reads were trimmed using Sickle v. 1.33 [9] to remove the base quality scores < 30 and lengths < 80 bp. The trimmed reads were corrected by RECKONER v. 1.1 [10]. KmerGenie v. 1.7044 [11] (with the parameter of “-k 141 --diploid”) and GenomeScope 2.0 [12] (with the parameter of “-m 10000”) were used to estimate the genome size of *N*. *mirabilis*. The *k*-mer frequencies used by GenomeScope were generated using Jellyfish 2.3.0 [13] with the parameters of “-s 5G” in the “count” step and “-h 3000000” for the “histo” step. Using the quality filtered WGS reads, the genome was assembled by Platanus 1.2.4 [14], Redundans 0.14a [15] (with the parameter of “--identity 0.9”), P_RNA_scaffolder [16] (with the parameter of “-e 1000000”), and Hapo-G 1.3.2 [17] (Table 1 Data file 1 Fig. 1 - assembling steps) [18]. After assembling, the assembled genome sequences were uploaded to GenBank for contamination assessment, and the potential contaminant sequences were then removed from the assembly. The repeat sequences of the contamination-free assembly were identified by RED v2.0 [19]. Using a repeat masked assembly, gene prediction and annotation were performed with the funannotate pipeline v1.8.13 [20], in which the “max_intronlen 1000000” was set for prediction. BUSCO 5.2.2 [21] was applied to perform a quality assessment of both the genome and transcriptome assemblies using the eudicots odb10-2020-09-10 database. Finally, from the assembled genome, the ploidy level of *N. mirabilis* was determined by nQuire using the “lrdmodel” function [22].

**Table 1:** Overview of all data files/data sets

Label	Name of data file/data set	File types (file extension)	Data repository and identifier (DOI or accession number)
Data file 1	Figure 1 A scheme chart to show the steps of genome assembly	Image file (.tif)	Figshare, 10.6084/m9.figshare.22336990 [18]
Data file 2	Raw WGS reads	Fastq file (.fastq)	NCBI Sequence Read Archive, https://identifiers.org/ncbi/ insdc.sra:SRR21047306 [23]
Data file 3	Raw RNA reads of leaf tissues	Fastq file (.fastq)	NCBI Sequence Read Archive, https://identifiers.org/ncbi/ insdc.sra:SRR21674160 [24]
Data file 4	Raw RNA reads of flower tissues	Fastq file (.fastq)	NCBI Sequence Read Archive, https://identifiers.org/ncbi/ insdc.sra:SRR21674159 [25]
Data file 5	Figure 2 Genome size estimation by a) KmerGenie, b-l) GenomeScope	Image file (.tif)	Figshare, 10.6084/m9.figshare.22336996 [26]
Data file 6	Transcriptome assembly	Fasta file (.fasta)	Figshare, 10.6084/m9.figshare.22336999 [27]
Data file 7	ORF- nucleotide sequences	Fasta file (.fasta)	Figshare, 10.6084/m9.figshare.22337008 [28]
Data file 8	ORF- translated sequences	Fasta file (.fasta)	Figshare, 10.6084/m9.figshare.22337200 [29]
Data file 9	BUSCO assessment of transcriptome assembly	Text (.txt)	Figshare, 10.6084/m9.figshare.22337203 [30]
Data file 10	Figure 3 Summary of GO annotation of open reading frames for the *Nepenthes mirabilis* transcriptome	Image file (.tif)	Figshare, 10.6084/m9.figshare.22337206 [31]
Data file 11	GO annotation of open reading frames for the *Nepenthes mirabilis* transcriptome	Text (.txt)	Figshare, 10.6084/m9.figshare.22337209 [32]
Data file 12	Pfam annotation of open reading frames for the *Nepenthes mirabilis* transcriptome	Text (.txt)	Figshare, 10.6084/m9.figshare.22337212 [33]
Data file 13	Assembled genome	Fasta file (.fasta)	NCBI Nucleotide, https://identifiers.org/nucleotide:JAODPA000000000.1 [34]
Data file 14	BUSCO assessment for genome assembly	Text (.txt)	Figshare, 10.6084/m9.figshare.22337215 [35]
Data file 15	Repeat annotation in the genome assembly	Text (.bed)	Figshare, 10.6084/m9.figshare.22337218 [36]
Data file 16	Predicted gene	Gff3 file (.gff3)	Figshare, 10.6084/m9.figshare.22337221 [37]
Data file 17	Predicted genes - nucleotide sequences	Fasta file (.fasta)	Figshare, 10.6084/m9.figshare.22337224 [38]
Data file 18	Predicted genes - translated sequences	Fasta file (.fasta)	Figshare, 10.6084/m9.figshare.22337227 [39]
Data file 19	Gene annotation using GO, Pfam, interPro and UniProt databases	Text (.txt)	Figshare, 10.6084/m9.figshare.22337230 [40]
Data file 20	Gene annotation from eggNOG-mapper analysis	Text (.txt)	Figshare, 10.6084/m9.figshare.22337236 [41]

The WGS produced ~ 139.5 GB of raw data (Data file 2) [23], while the RNA-seq produced ~ 21.7 Gb of raw data for leaf tissues (Data file 3) [24] and ~ 27.9 GB of raw data for flower tissues (Data file 4) [25]. KmerGenie estimated the genome size of *N. mirabilis* at 791,766,648 bp under the best selected *k*-mer of 99, following a comparison of different *k*-mer spectra. GenomeScope estimated it between 640,605,912 and 776,677,804 bp using a *k*-mer from 17 to 99 (Table 1 Data file 5 Fig.2 - genome size estimation) [26]. GenomeScope also revealed that the level of heterozygosity in *N. mirabilis* was between 0.3% and 1%; the repeat content ranged from 13.8% at 99-mer to 50.4% at 17-mer.

The TransPi pipeline assembled a total of 339,802 transcripts (Data file 6) [27], with the longest transcript being 25,441 bp and an N50 of 945 bp. 79,758 ORFs were obtained in the transcriptome assemblies (Data files 7 and 8) [28, 29], of which 55,993 were complete. The BUSCO assessment for the ORFs indicated 93.7% completeness (Data file 9) [30]. The GO annotations for all ORFs revealed that they were mainly functionally related to proteolysis and DNA integration in the ‘Biological Processes’ category (Table 1 Data file 10 Fig.3 - GO annotation of open reading frames ) [31]. The full annotation of GO and Pfam for ORFs can be found in Data files 11 and 12 [32, 33].

The genome assembly was 691,409,685 bp with 159,555 contigs/scaffolds and an N50 of 10,307 bp (Data file 13) [34]. The largest scaffold was 312,611 bp, and the shortest was 200 bp. The BUSCO assessment for the assembly indicated 91.1% completeness (Data file 14) [35]. The ploidy level estimated by nQuire indicated a diploid genome of *N. mirabilis*, as the diploid model displayed the lowest delta likelihood from the free model (diploid delta likelihood: 626,726.449; triploid delta likelihood: 1,581,220.976; tetraploid delta likelihood: 1,425,949.675). Repeat prediction identified 44.21% (305,683,241 bp) of the genome as repetitive regions (Data file 15) [36]. Funannotate predicted 42,961 genes that code for 45,461 proteins (Data files 16–18) [37–39]. The gene annotation is shown in Data files 19–20 [40, 41].

## Limitations

The assembled genome is still fragmented and not suitable for genome structure analysis. Further high-quality genome assemblies using long read, Hi-C, and other sequencing technologies are needed.

In particular, it is currently a challenge to obtain an accurate genome size with only short whole genome sequencing reads [42, 43], which is also true for *N. mirabilis* in this study. Although both KmerGenie and GenomeScope estimated comparable genome sizes at a *k*-mer of 99 with 791,766,648 bp and 774,029,535 bp, respectively, we observed that the estimated genome size was affected by increasing *k*-mer from 17 to 71 in GenomeScope [26]. Considering that a high *k*-mer might introduce errors in genome size estimation [12], a genome size of 99-mer could be misestimated. At small *k*-mers of 17 and 21, the genome size by GenomeScope was 640,605,912 bp and 675,699,094 bp, respectively. These results are then comparable to the final assembled genome size of 691,409,685 bp in *N. mirabilis*. Nevertheless, long HiFi sequencing provides an alternative way to use a larger *k*-mer and circumvent sequence errors in characterizing the species genome size and other features accurately [44].

In this study, RED [19] was used for repeat sequence identification, given its fastness, efficiency and accuracy in repeat detection [45, 46]. Compared to repeat content estimation by GenomeScope, whose results varied dramatically among different *k*-mers which made its results less referable, RED results provided an initial guideline for the percentage of repeat content in *N. mirabilis*. However, considering the fragmented nature of the assembly, repeat regions might still be missing and misassembled. Moreover, RED does not classify repeats into different types (e.g. retrotransposons, DNA transposons, and so on), additional repeat detectors are needed if the evolutions of different repeats are under investigation [47].

## Data Availability

We deposited the raw sequenced reads to the NCBI Sequence Read Archive under accession number SRR21047306 for whole genome sequencing reads (https://identifiers.org/ncbi/ insdc.sra:SRR21047306) [23], SRR21674160 for leaf RNA-seq reads (https://identifiers.org/ncbi/ insdc.sra:SRR21674160) [24], SRR21674159 for flower RNA-seq reads (https://identifiers.org/ncbi/ insdc.sra:SRR21674159) [25], and JAODPA000000000 for the assembled genome (https://identifiers.org/nucleotide:JAODPA000000000.1) [34]. Genome/transcriptome assemblies are also available at 10.6084/m9.figshare.21252198. Please further see Table 1 for details and references [1–22, 26–33, 35–41] of the results of genome and transcriptome analyses submitted to figshare.
